# CRISPR/Cas9-mediated mutation in auxin efflux carrier *OsPIN9* confers chilling tolerance by modulating reactive oxygen species homeostasis in rice

**DOI:** 10.3389/fpls.2022.967031

**Published:** 2022-08-01

**Authors:** Huawei Xu, Xiaoyi Yang, Yanwen Zhang, Huihui Wang, Shiyang Wu, Zhuoyan Zhang, Golam Jalal Ahammed, Chunzhao Zhao, Hao Liu

**Affiliations:** ^1^College of Agriculture, Henan University of Science and Technology, Luoyang, China; ^2^College of Horticulture and Plant Protection, Henan University of Science and Technology, Luoyang, China; ^3^Shanghai Center for Plant Stress Biology, CAS Center for Excellence in Molecular Plant Sciences, Chinese Academy of Sciences, Shanghai, China

**Keywords:** auxin efflux carrier, CRISPR/Cas9, *Oryza sativa*, *OsPIN9*, chilling tolerance

## Abstract

Phytohormone auxin plays a vital role in plant development and responses to environmental stresses. The spatial and temporal distribution of auxin mainly relies on the polar distribution of the PIN-FORMED (PIN) auxin efflux carriers. In this study, we dissected the functions of *OsPIN9*, a monocot-specific auxin efflux carrier gene, in modulating chilling tolerance in rice. The results showed that *OsPIN9* expression was dramatically and rapidly suppressed by chilling stress (4°C) in rice seedlings. The homozygous *ospin9* mutants were generated by CRISPR/Cas9 technology and employed for further research. *ospin9* mutant roots and shoots were less sensitive to 1-naphthaleneacetic acid (NAA) and *N*-1-naphthylphthalamic acid (NPA), indicating the disturbance of auxin homeostasis in the *ospin9* mutants. The chilling tolerance assay showed that *ospin9* mutants were more tolerant to chilling stress than wild-type (WT) plants, as evidenced by increased survival rate, decreased membrane permeability, and reduced lipid peroxidation. However, the expression of well-known *C-REPEAT BINDING FACTOR* (*CBF*)/*DEHYDRATION-RESPONSIVE ELEMENT-BINDING PROTEIN 1* (*DREB*)-dependent transcriptional regulatory pathway and Ca^2+^ signaling genes was significantly induced only under normal conditions, implying that defense responses in *ospin9* mutants have probably been triggered in advance under normal conditions. Histochemical staining of reactive oxygen species (ROS) by 3′3-diaminobenzidine (DAB) and nitroblue tetrazolium (NBT) showed that *ospin9* mutants accumulated more ROS than WT at the early stage of chilling stress, while less ROS was observed at the later stage of chilling treatment in *ospin9* mutants. Consistently, antioxidant enzyme activity, including catalase (CAT), peroxidase (POD), and superoxide dismutase (SOD), improved significantly during the early chilling treatments, while was kept similar to WT at the later stage of chilling treatment, implying that the enhanced chilling tolerance of *ospin9* mutants is mainly attributed to the earlier induction of ROS and the improved ROS scavenging ability at the subsequent stages of chilling treatment. In summary, our results strongly suggest that the *OsPIN9* gene regulates chilling tolerance by modulating ROS homeostasis in rice.

## Introduction

Due to their sessile nature, plants have to cope with various abiotic stresses, such as chilling stress. Chilling stress affects plant growth and development and restricts geographical distribution (Shi et al., 2018; Ding et al., 2020). Rice (*Oryza sativa* L.) is sensitive to chilling stress (Wang et al., 2003), and extreme temperature is a crucial factor limiting rice plant distribution and impairing rice quality and yield (Imin et al., 2004; Zeng et al., 2017). Therefore, improving rice chilling tolerance for maintaining rice yield is an urgent target by molecular genetic tools (Ma et al., 2015).

In recent years, the mechanism of plant adaption to cold stress has been extensively studied. Ca^2+^ influx, triggered by chilling stress, plays a crucial role in inducing temperature-responsive gene expression (Knight et al., 1996; Gong et al., 1998; Zhu, 2016; Ding et al., 2020). COLD1, a G-protein regulator, interacts with RGA1 (RICE G-PROTEIN α SUBUNIT 1) to activate the Ca^2+^ channel to improve chilling tolerance (Ma et al., 2015). Additionally, CBL-INTERACTING PROTEIN KINASE 7 (OsCIPK7) and CYCLIC NUCLEOTIDE-GATED CHANNELS 9 (OsCNGC9) were also shown to mediate Ca^2+^ influx under chilling stress in rice (Zhang et al., 2019a; Wang et al., 2021b). Growing evidence shows that the well-known *C-REPEAT BINDING FACTOR* (*CBF*)*/DEHYDRATION-RESPONSIVE ELEMENT-BINDING PROTEIN 1* (*DREB*)-dependent transcriptional regulatory genes are dramatically induced by low temperature and may play a vital role in plant chilling tolerance (Stockinger et al., 1997; Liu et al., 1998; Ding et al., 2019; Sun et al., 2021), whereas reports also showed that there is no direct connection between the transcriptional abundance of these transcription factors and the degree of cold tolerance (Mao and Chen, 2012; Zhao et al., 2015; Zhang et al., 2016). By contrast, reactive oxygen species (ROS) homeostasis, including ROS induction and scavenging, was demonstrated to play a crucial role in chilling tolerance (Lee et al., 2002; Dong et al., 2009; Du et al., 2013b; Zhang et al., 2016).

The phytohormone auxin (indole-3-acetic acid, IAA) is essential in many plant developmental processes, including organ formation, vascular differentiation, apical dominance, flowering, and many other physiological processes (Benkova et al., 2003; Friml, 2003; Mattsson et al., 2003; Leyser, 2006; Zhao et al., 2013). In addition, auxin also participates in response to many environmental signals, such as light, gravity, and cold (Friml, 2003; Kimura and Kagawa, 2006; Palme et al., 2006; Shibasaki et al., 2009; Du et al., 2013a). Auxin mainly synthesizes in aerial organs (Woodward and Bartel, 2005; Zhao, 2008, 2010), such as leaf primordial and young leaves, and is transported basipetally by several transporters, which at least including the AUXIN1 (AUX1)/LIKE AUX1 (LAX) influx carriers, the PHOSPHOGLYCOPROTEIN (PGP/MDR/ABCB) efflux/influx transporters, and the PIN-FORMED (PIN) auxin efflux carriers (Michniewicz et al., 2007). Among which, PIN family proteins were reported to play a vital role in this process (Petrasek et al., 2006).

Although auxin has been proved to play an essential role in modulating almost all aspects of plant growth and development, its underlying mechanism under cold stress is elusive. Increasing evidence implied that cold stress is linked to auxin homeostasis. Transcript profiling showed that many auxin-responsive genes were differentially expressed under cold stress (Jain and Khurana, 2009), and cold treatment significantly increased IAA content in rice leaves (Du et al., 2013a). Auxin homeostasis is closely related to *GH3* genes encoding auxin-conjugating enzymes. In *Arabidopsis*, overproduction of a *GH3* gene *WES1* by insertion mutation resulted in decreased auxin content and enhanced cold tolerance (Park et al., 2007). Consistently, overexpression of a rice *GH3* family gene, *OsGH3-2,* and disruption of ABA and IAA biosynthesis gene also caused decreased IAA content and increased cold tolerance in rice (Du et al., 2012, 2013b), indicating that auxin homeostasis plays a potential role in regulating cold tolerance. In addition, auxin transport was shown to implicate in chilling response. An earlier report showed that the velocity of exogenous auxin transport was affected by temperature in some plant species (Morris, 1979). Cold treatment inhibited auxin transport basipetally, while room temperature restored it (Nadella et al., 2006). Further research demonstrated that cold stress affected auxin homeostasis by inhibition of intracellular trafficking of auxin efflux carriers (Shibasaki et al., 2009), and GNOM, a SEC7 containing ARF-GEF, which links to endosomal trafficking of auxin efflux carriers, was reported to positively modulate cold tolerance in *Arabidopsis* (Ashraf and Rahman, 2019). Collectively, these reports demonstrated that auxin homeostasis and transport play a potential role in regulating plant chilling tolerance. However, there is limited information on the role of auxin homeostasis and transport in chilling stress and the underlying molecular mechanisms.

Previous reports have shown that there are 12 *PIN* genes in the rice genome according to their relationship with PIN proteins reported in *Arabidopsis*, among which, *OsPIN9*, *OsPIN10a*/*3a,* and *OsPIN10b*/*3b* were identified as three monocot-specific *PIN* genes (Wang et al., 2009). It was demonstrated that OsPIN9 is a plasma membrane protein and robustly expressed in the vascular tissue of the roots and junctions; modulating the expression of *OsPIN9* can change the transport and distribution of auxin (Hou et al., 2021). Our previous research showed that mutation of *OsPIN9* by CRISPR/Cas9 significantly decreased the shoot height and adventitious root number at the seedling stage (Wu et al., 2021). However, little is known about the role of *OsPIN9* in chilling stress response and tolerance in plants. Here, we examined the function of *OsPIN9* in modulating chilling tolerance by CRISPR/Cas9 technology. Our results showed that chilling stress quickly and dramatically decreased the expression of *OsPIN9*, and mutation of *OsPIN9* led to enhancement of chilling tolerance in rice. The ROS, accumulated at the early chilling stage in *ospin9* mutants, probably as a signal to trigger the antioxidant enzyme system to quench the subsequent oxidative burst at the later chilling stage and contribute to the improved chilling tolerance. These results proved that mutation of *OsPIN9* can substantially improve chilling tolerance and shed light on the relationship between *OsPIN9*-mediated polar auxin transport and chilling tolerance in rice.

## Materials and methods

### Growth conditions and stress treatments

Rice (*O. sativa* L.) seeds of Nipponbare were sterilized with 3% NaOCl for 30 min, washed with distilled water for 3–5 times, and incubated in Petri dishes with wetted filter papers at 30°C for 2–3 days in the dark. Then, germinated seeds were transferred to Kimura B complete nutrient solution (Xu et al., 2006) under greenhouse conditions (temperature of 30/25°C (day/night), relative humidity 60%–80%, light intensity 300 μmol/m^2^/s, and photoperiod of 12 h day/12 h night).

Fourteen-day-old seedlings cultured in the Kimura B solution were employed for all stress treatments. For drought treatment, seedlings were treated with 20% PEG; for salt treatment, seedlings were cultured in 10 g/L (w/v) NaCl; for chilling treatment, seedlings were transferred to 4°C conditions for 4 or 6 days, and then transferred back to the greenhouse at 28°C and grow for another 4 days to analyze the recovery and survival rate. Leaves from Wild-type (WT) and *ospin9* mutants were collected at the indicated time points and used for physiological analysis.

### RNA extraction and quantitative real-time PCR (qRT-PCR) analysis

The specific primer pairs were designed for the quantitative RT-PCR (qRT-PCR) analysis (Supplementary Table 1). Total RNA isolation was performed using RNAiso Plus (TAKARA BIO INC). RNA concentration, purity, and integrity were assessed with a NanoDrop 2000 spectrophotometer (Thermo Fisher Scientific, United States), and electrophoretic separation in 1% agarose gel. cDNA synthesis was finished using HiScript III RT SuperMix for qRT-PCR (+gDNA wiper; Nanjing Vazyme Biotech Company, Ltd.). qRT-PCR was conducted using AceQ Universal SYBR qPCR Master Mix (Nanjing Vazyme Biotech Company, Ltd.) and Lightcycler^®^ 96 system. Three biological replicates and three technical repetitions were carried out to detect the gene expression. The specificity of amplified PCR products was verified by melting curve analysis. The data were normalized to the amplification of the *OsACTIN1* gene (Os03g0718100). For abiotic stress treatments, the expression level of the *OsPIN9* gene in CK was defined as 1.

### Mutants generation and identification

The gene-editing vector CRISPR-RICE was used for the creation of *ospin9* mutants in our previous report (Wu et al., 2021). Three homozygous *ospin9* mutants, *ospin9-1 ospin9-2,* and *ospin9-3*, were identified according to the previous report (He et al., 2019). For off-target analysis, a toolkit for CRISPR-based genome editing CRISPR-GE^1^ was employed to analyze the potential off-target sites, and the genotype of targeted mutation was confirmed by sequencing of the PCR products directly (Liu et al., 2015). All primers used in this study were listed in Supplementary Table 1.

### Phenotypic analysis and 1-naphthaleneacetic acid or *N*-1-naphthylphthalamic acid treatment

Seven-day-old and 14-day-old seedlings were used for phenotype analysis at the seedling stage. The agronomic traits, such as plant height, tiller number, and panicle length, were collected at the mature stage. For NAA or NPA treatment, germinated seeds were transferred to a nutrient solution containing 0.1 μM NAA or 0.5 μM NPA for 7 days, and then, the shoot height and root length were analyzed. WT and *ospin9* mutants cultured under normal conditions were used as a control in this study.

### Physiological analysis and histochemical staining

Trypan blue staining was performed according to Choi et al. (2007). Electrolyte conductivity and malondialdehyde (MDA) contents were measured as described previously (Hu et al., 2020). 3′3-diaminobenzidine (DAB) staining and nitroblue tetrazolium (NBT) staining were conducted as previously reported (Zhang et al., 2016). Catalase (CAT), peroxidase (POD), and superoxide dismutase (SOD) activities were measured by the methods of Wu et al. (2003). Protein content was tested by Coomassie Brilliant Blue G-250 staining method (Bradford, 1976).

### Statistical analysis

All experiments were repeated at least three times with consistent results. All data were given as means ± SD and analyzed by one-way analysis of variance (ANOVA) method in GraphPad PRISM (8.0.2) at the significance levels of *p* < 0.05 (^*^), *p* < 0.01 (^**^) and *p* < 0.001 (^***^).

## Results

### Chilling stress rapidly and dramatically suppresses *OsPIN9* expression

**Figure 1:**
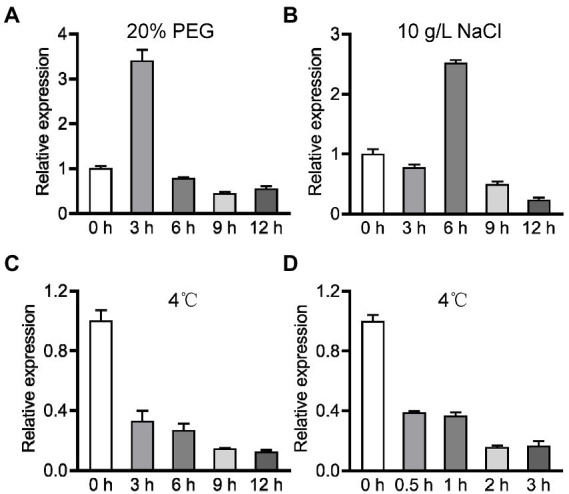
Expression profile analysis of *OsPIN9* under various abiotic stresses. **(A)** 20% PEG; **(B)** 10 g/L NaCl; **(C)** 4°C treated for 0–12 h. **(D)** 4°C treated for 0–3 h.

To dissect the role of *OsPIN9* in abiotic stress, we performed qRT-PCR to analyze the expression of *OsPIN9* under drought, salt, and chilling treatments. *OsPIN9*, which showed a similar expression profile under drought and salt treatments, was firstly induced greatly and then repressed rapidly in rice roots (Figures 1A,B). In contrast to drought and salt treatments, *OsPIN9* transcriptional abundance dramatically decreased by about 67% in rice roots after being transferred to chilling conditions for 3 h, and the abundance progressively decreased by about 90% after chilling for 12 h (Figure 1C). To determine whether *OsPIN9* is an early chilling-regulated gene, we further investigated the expression of *OsPIN9* at a shorter chilling treatment time. The time-course analyses of chilling treatment indicated that chilling treatment rapidly repressed the expression of *OsPIN9* even after being transferred to chilling conditions only for half an hour (Figure 1D). These results indicate that *OsPIN9* is rapidly inhibited by chilling treatment, implying a potential role of *OsPIN9* in regulating chilling tolerance.

### Generating *ospin9* mutants *via* CRISPR/Cas9 and phenotypes of transformants

**Figure 2:**
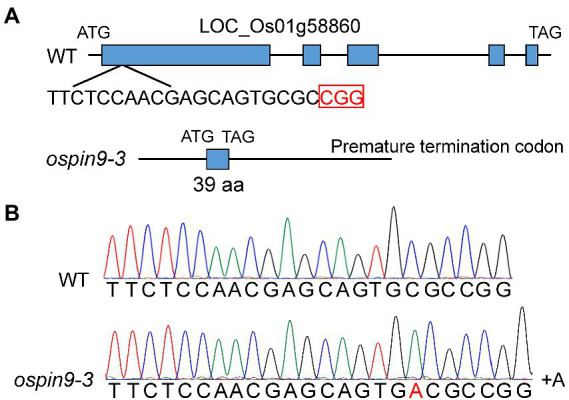
Generation of *ospin9* mutants. **(A)** Gene structure of WT and *ospin9* mutants. Blue box indicates the *OsPIN9* gene region. **(B)** One base-pair nucleotide A was inserted in the *ospin9-3* mutant.

To investigate the function of *OsPIN9*, we have employed the CRISPR/Cas9 technology to generate two *ospin9* mutants, *ospin9-1* and *ospin9-2* (Wu et al., 2021). The identities of amino acids of OsPIN9 proteins in these two mutants predicted based on the nucleotide sequences was up to 99% and only one amino acid is different (Wu et al., 2021). To confirm that the phenotype of *ospin9* mutants was indeed caused by the mutation of *OsPIN9*, we further obtained another *ospin9* homozygous mutant (*ospin9-3*), which contained an A base insertion at the 18th base of the target sequence and caused premature termination of translation (Figure 2). *ospin9-3* owned a shorter OsPIN9 protein (39 aa) compared with *ospin9-1* and *ospin9-2* (172 aa). The potential off-target sequence of *OsPIN9* was further predicted in the rice genome and no nucleotide change was detected (data not shown). The homozygous mutants of *ospin9-1* and *ospin9-3* were used for further experiments.

The previous study showed that mutation of *OsPIN9* affects rice architecture (Hou et al., 2021). We evaluated the phenotype of *ospin9-1* and *ospin9-3* at the seedling stage. Consistent with previous report (Hou et al., 2021), mutation of *OsPIN9* significantly decreased shoot height, root length, and adventitious root number at the seedling stage (Supplementary Figure 1), suggesting that *OsPIN9* plays a vital role in regulating plant architecture.

### The disturbance of auxin homeostasis in *ospin9* mutants

**Figure 3:**
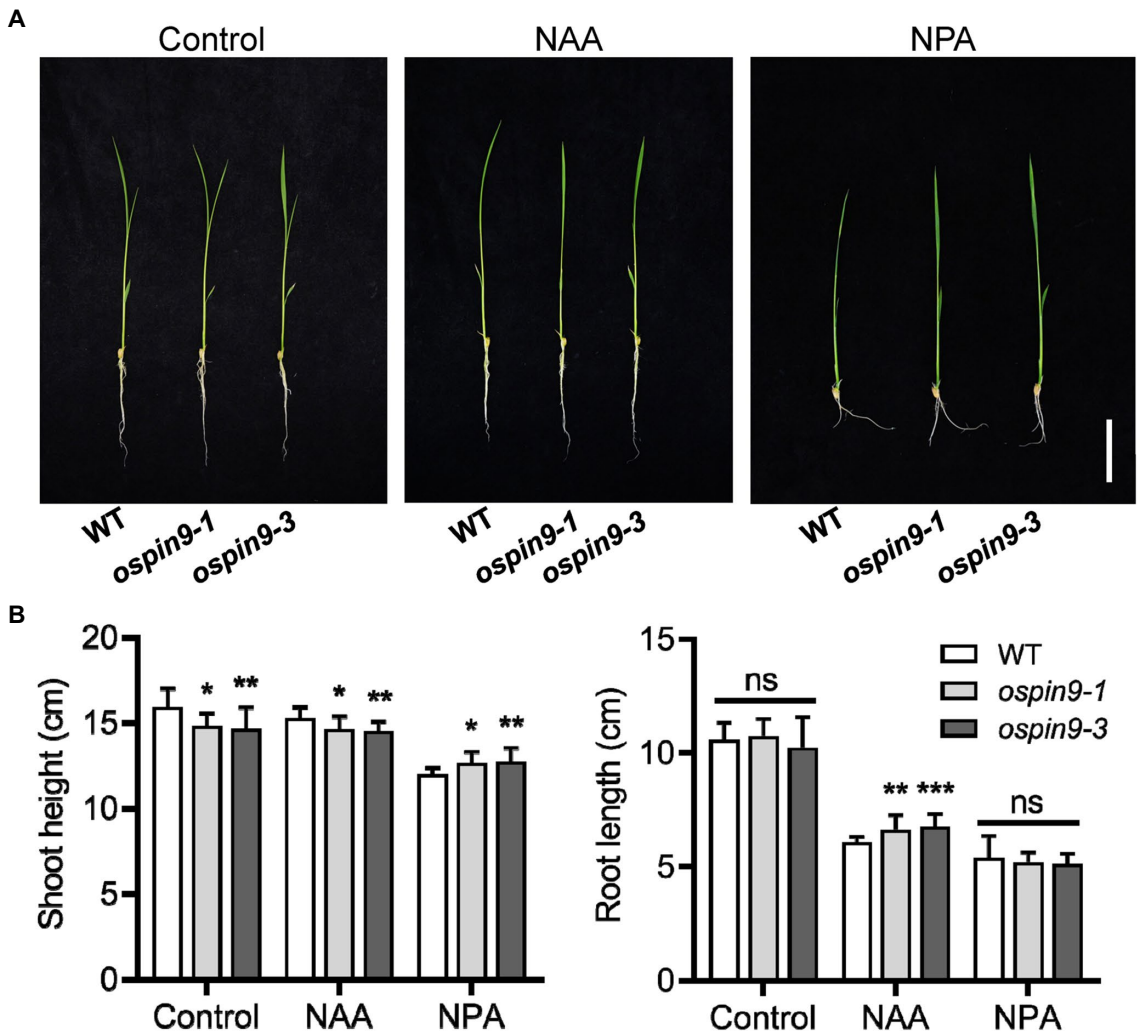
Response of WT and *ospin9* mutants to NAA and NPA treatment. **(A)** Phenotype of WT and *ospin9* mutants under normal, NAA or NPA treatment. Bar = 5 cm. **(B)** Shoot height and root length of WT and *ospin9* mutants under normal, NAA or NPA treatment. Values are means ± standard deviation (SD; *n* = 15). Data were analyzed by ANOVA and Tukey’s tests at *p* < 0.05 significant level.

1-Naphthaleneacetic acid can enter into cells by passive diffusion and be employed to study the sensitivity to auxin in various auxin-related mutants (Delbarre et al., 1996; Li et al., 2019). *N*-1-naphthylphthalamic acid is a classical inhibitor of polar auxin transport in plants (Abas et al., 2021). We assayed the effects of mutation of *OsPIN9* on auxin homeostasis with NAA or NPA treatment in WT and *ospin9* mutants. Germinated seeds were transferred to NAA or NPA solution for 7 days, and then, the shoot height and root length were assessed. 1-Naphthaleneacetic acid and NPA treatment significantly increased the primary root length and shoot height of *ospin9* mutants compared with WT plants, respectively (Figure 3), indicating that the roots and shoots of *ospin9* were less sensitive to NAA and NPA treatment, respectively. These results demonstrate that auxin homeostasis in *ospin9* mutants is disrupted.

### Mutation of *OsPIN9* improves the chilling tolerance in rice

**Figure 4:**
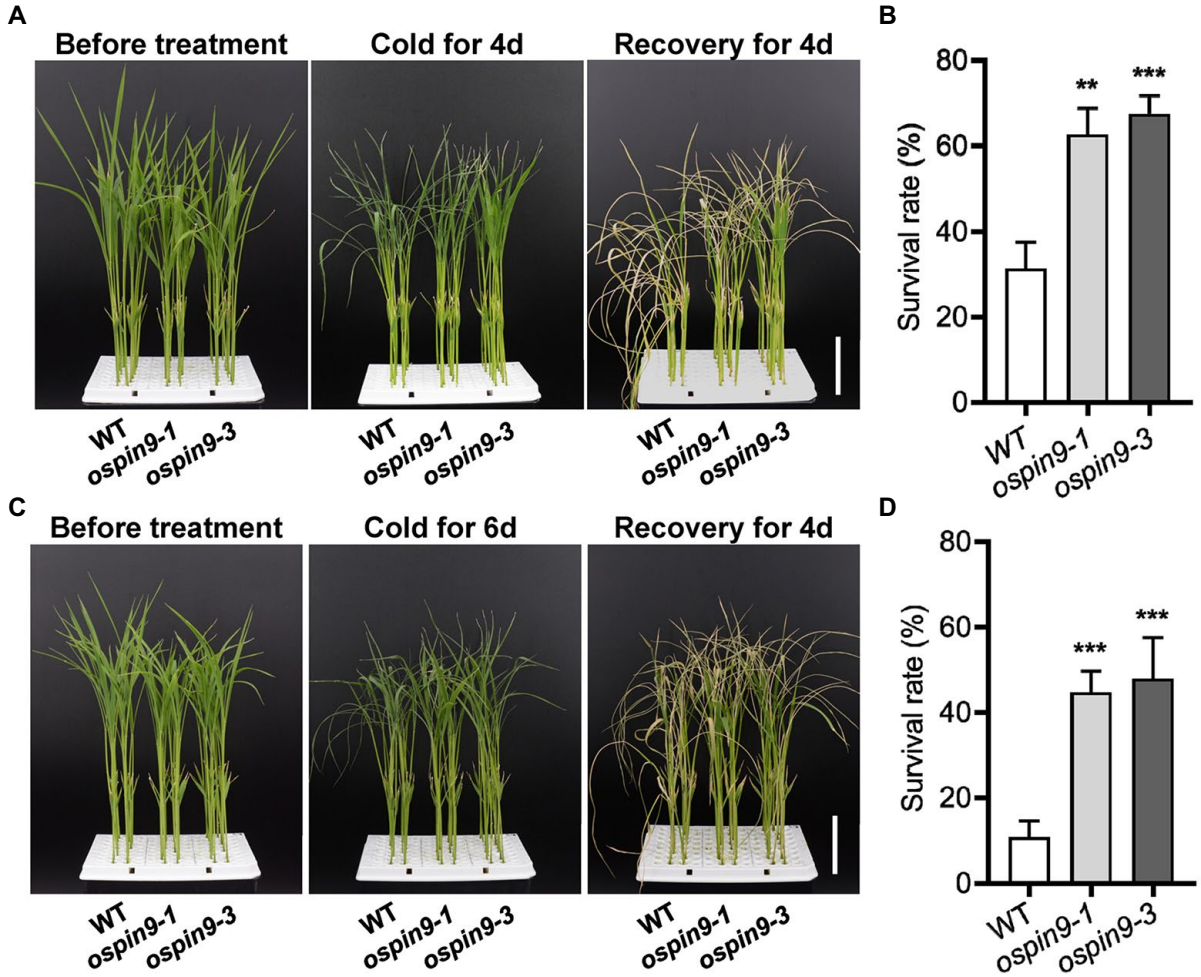
Mutation of *OsPIN9* enhanced chilling tolerance in rice. **(A)** Phenotype of WT and *ospin9* mutants before chilling treatment, after chilling treatment for 4 days and recovery for another 4 days. Bar = 4 cm. **(B)** Statistical presentation of survival rate after chilling 4 days and recovery for another 4 days. **(C)** Phenotype of WT and *ospin9* mutants before chilling treatment, after chilling treatment for 6 days and recovery for another 4 days. Bar = 4 cm. **(D)** Statistical presentation of survival rate after chilling 6 days and recovery for another 4 days. Values are means ± standard deviation (SD; *n* ≥ 30). Data were analyzed by ANOVA and Tukey’s tests at *p* < 0.05 significant level.

Since *OsPIN9* was inhibited quickly by chilling treatment (Figures 1C,D), the role of *OsPIN9* in chilling tolerance was evaluated in *ospin9* mutants and WT plants. After chilling for 4 days, almost all of the leaves of WT plants showed rolling and wilting, while part of *ospin9* leaves remained normal (Figure 4A), and *ospin9-1* and *ospin9-3* showed 62.5% and 67.4% survival rates, respectively, compared to 31.3% in WT plants after another 4 days of recovery at 28°C (Figure 4B). After 6 days of chilling treatment, there was no visual difference between WT and *ospin9* mutant leaves (Figure 4C), and the survival rate of *ospin9* mutants was still remarkably higher than that of WT after another 4 days of recovery (Figure 4D). These results suggest that *OsPIN9* is involved in the modulation of chilling stress alleviation.

**Figure 5:**
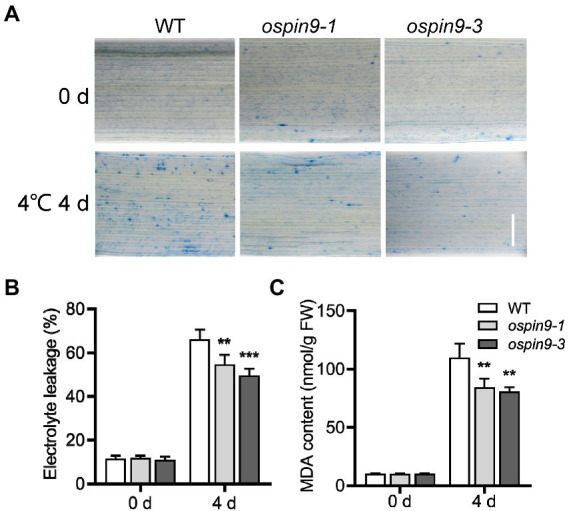
Mutation of *OsPIN9* altered cell damage, cell membrane stability and lipid peroxidation in rice seedling leaves. **(A)** Trypan blue staining; Bar = 1 mm. **(B)** Electrolyte leakage; **(C)** Malondialdehyde contents. The samples were taken randomly from the leaves of at least six plants of each independent (WT, *ospin9-1*, and *ospin9-3*) line after different time points of chilling stress. Values are means ± standard deviation (SD; *n* = 6). Data were analyzed by ANOVA and Tukey’s tests at *p* < 0.05 significant level.

Cold stress causes cell damage (Ding et al., 2019). We further evaluated the cell damage by trypan blue staining for cell death, electrolyte leakage, and MDA contents for membrane integrity. There was no apparent difference in trypan blue staining between WT and *ospin9* mutant leaves under normal growth conditions, while staining with trypan blue was more intense in leaves from WT plants compared to the *ospin9* mutants after chilling for 4 days (Figure 5A), indicating that the cell death in *ospin9* is less than that in WT plants. Cold stress often damages cell membranes, and electrolyte leakage is usually a representative of damage to cell membranes by cold stress (Du et al., 2012); therefore, electrolyte leakage was assessed. The electrolyte leakage of *ospin9* mutants was consistent with WT before chilling treatment. However, it was significantly lower in *ospin9* mutants than that in WT plants after chilling for 4 days (Figure 5B). Malondialdehyde contents, derived from lipid peroxidation of polyunsaturated fatty acid, were similar to WT before chilling treatment while markedly decreased in *ospin9* leaves than WT plants after chilling treatment (Figure 5C). Taken together, these results strongly demonstrate that *ospin9* mutants are more resistant to chilling stress than WT plants and the mutation of *OsPIN9* contributes to the enhanced chilling tolerance in *ospin9* mutants.

### The role of CBF/DREB regulon and Ca^2+^ signaling in regulating chilling tolerance in *ospin9* mutants

**Figure 6:**
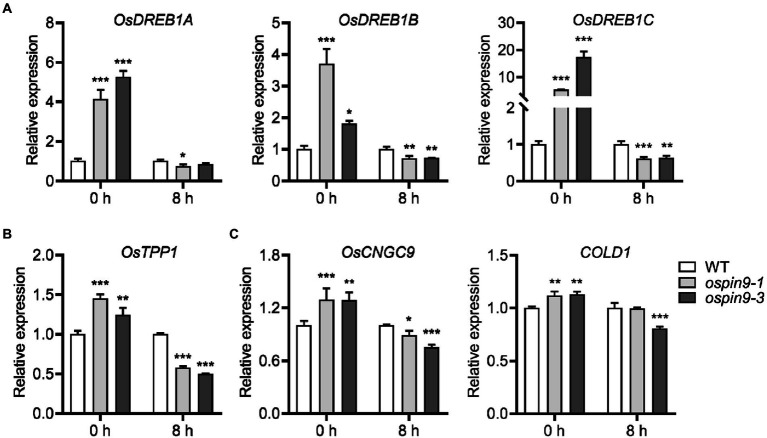
*OsDREB1* genes, *OsTPP1,* and Ca^2+^ signaling genes expression under normal and chilling conditions in WT and *ospin9* mutants. **(A)** *OsDREB1s* gene expression analysis. **(B)** *OsTPP1* expression analysis. **(C)** Ca^2+^ signaling genes expression analysis. Values were means ± standard deviation (SD; *n* = 3). Data were analyzed by ANOVA and Tukey’s tests at *p* < 0.05 significant level.

Over the past two decades, a series of components have been identified in cold-stress signaling pathways, such as the well-known CBF/DREB regulon and Ca^2+^ signaling (Zhang et al., 2016; Ding et al., 2020). To further investigate the underlying mechanism of *OsPIN9* in chilling stress, we firstly performed qRT-PCR to evaluate whether the *CBF/DREB* genes were related to the improved chilling tolerance in *ospin9* mutants. *OsDREB1A*, *OsDREB1B*, and *OsDREB1C* expressions were analyzed due to the early induction by cold in rice (Dubouzet et al., 2003; Mao and Chen, 2012). Under normal conditions, these three *OsDREB1* genes in *ospin9* mutants showed a significantly higher accumulation than WT plants, while the induction level of these *OsDREB1* genes in *ospin9* mutants changed to a similar even lower level compared to WT plants after chilling for 8 h (Figure 6A). In addition, it was demonstrated that MITOGEN-ACTIVATED PROTEIN KINASE3 (OsMAPK3) phosphorylates OsbHLH002/INDUCER of CBF EXPRESSION1 (OsICE1) and promotes *TREHALOSE-6-PHOSPHATE PHOSPHATASE1* (*OsTPP1*) expression to improve chilling tolerance in rice (Zhang et al., 2017). Consistent with the expression of *OsDREB1* genes, *OsTPP1* was markedly induced under normal conditions rather than under chilling conditions (Figure 6B). Besides the well-known CBF/DREB regulon, Ca^2+^ signaling-related genes, such as *COLD1* and *OsCNGC9*, also play a vital role in regulating plant cold tolerance (Ma et al., 2015; Wang et al., 2021b). Similar to the expression of *OsDREB1* and *OsTPP1* genes, the expression of *COLD1* and *OsCNGC9* was also significantly increased in *ospin9* mutants compared with WT plants under normal conditions. After being transferred to low-temperature conditions for 8 h, the expression showed a similar or decreased level than that in WT plants (Figure 6C). Collectively, these results indicated that mutation of *OsPIN9* probably triggers the induction of plant defense response in advance under normal conditions, which may contribute to the enhanced chilling tolerance under chilling stress, while these genes did not show a striking increase after chilling for 8 h, implying that CBF/DREB regulon might play a minor role in modulating *ospin9* chilling tolerance under chilling conditions. Considering that Ca^2+^ signaling plays a role during short-term cold treatments (Ma et al., 2015; Wang et al., 2021b), the role of these genes in regulating *ospin9* chilling responses still needs further investigation.

### ROS homeostasis contributes to the higher chilling tolerance of *ospin9* mutants under chilling stress

**Figure 7:**
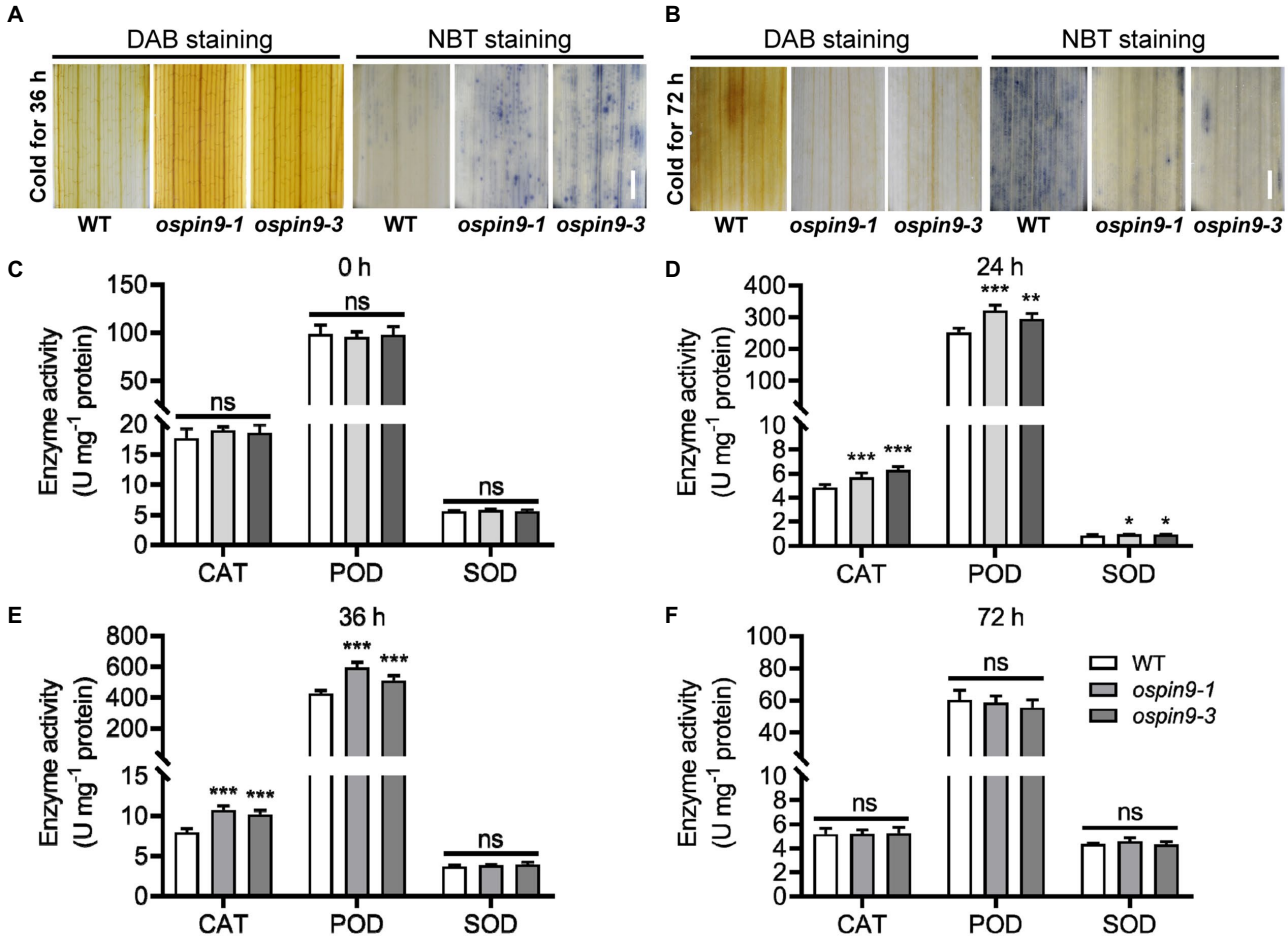
ROS production and scavenging in WT and *ospin9* mutants during chilling stress. **(A)** ROS accumulation in WT and *ospin9* mutants was detected at 36 h with NBT and DAB staining. Bar = 1 mm. **(B)** DAB and NBT staining for WT and *ospin9* mutants after chilling treatment for 72 h. Bar = 1 mm. **(C–F)** Antioxidant enzyme activities analysis after chilling for 0, 24, 36, and 72 h in WT and *ospin9* mutants. Values were means ± standard deviation (SD; *n* = 6). Data were analyzed by ANOVA and Tukey’s tests at *p* < 0.05 significant level.

Cold stress induces ROS production (Zhang et al., 2016; Guo et al., 2018). Low-level ROS at the early stress stage can be signaled to trigger stress-responsive activities (Zhang et al., 2019b), so we investigated the ROS content by DAB staining for hydrogen peroxide (H_2_O_2_) and NBT staining for superoxide anion radicals ( $O_{2}^{-}$ ) under chilling conditions. After chilling for 36 h, the *ospin9* leaves accumulated more ROS than WT (Figure 7A). In contrast, after chilling for 72 h, strong ROS accumulation was only observed in WT plants, whereas feeble levels of ROS were detected in *ospin9* mutants (Figure 7B). The antioxidant enzyme system, such as SOD, CAT, and POD, has been proved to function in quenching ROS under various adverse conditions (Jaleel et al., 2009; Shah et al., 2021; Song et al., 2021). We then analyzed the activity of these three enzymes under normal and chilling conditions. The results showed that the activity of CAT, POD, and SOD was comparable with WT plants under normal conditions (Figure 7C), while it was significantly higher than that of WT plants after chilling for 24 h (Figure 7D), and after chilling for 36 h, SOD activity was comparable with WT, while the activity of CAT and SOD was still noticeably higher than that of WT (Figure 7E). This increase was completely abolished after chilling for 72 h between *ospin9* mutants and WT plants (Figure 7F). These results indicate that ROS homeostasis, including ROS production and scavenging, plays a vital role in modulating chilling tolerance of *ospin9* mutants.

### IAA triggers the production of H_2_O_2_

**Figure 8:**
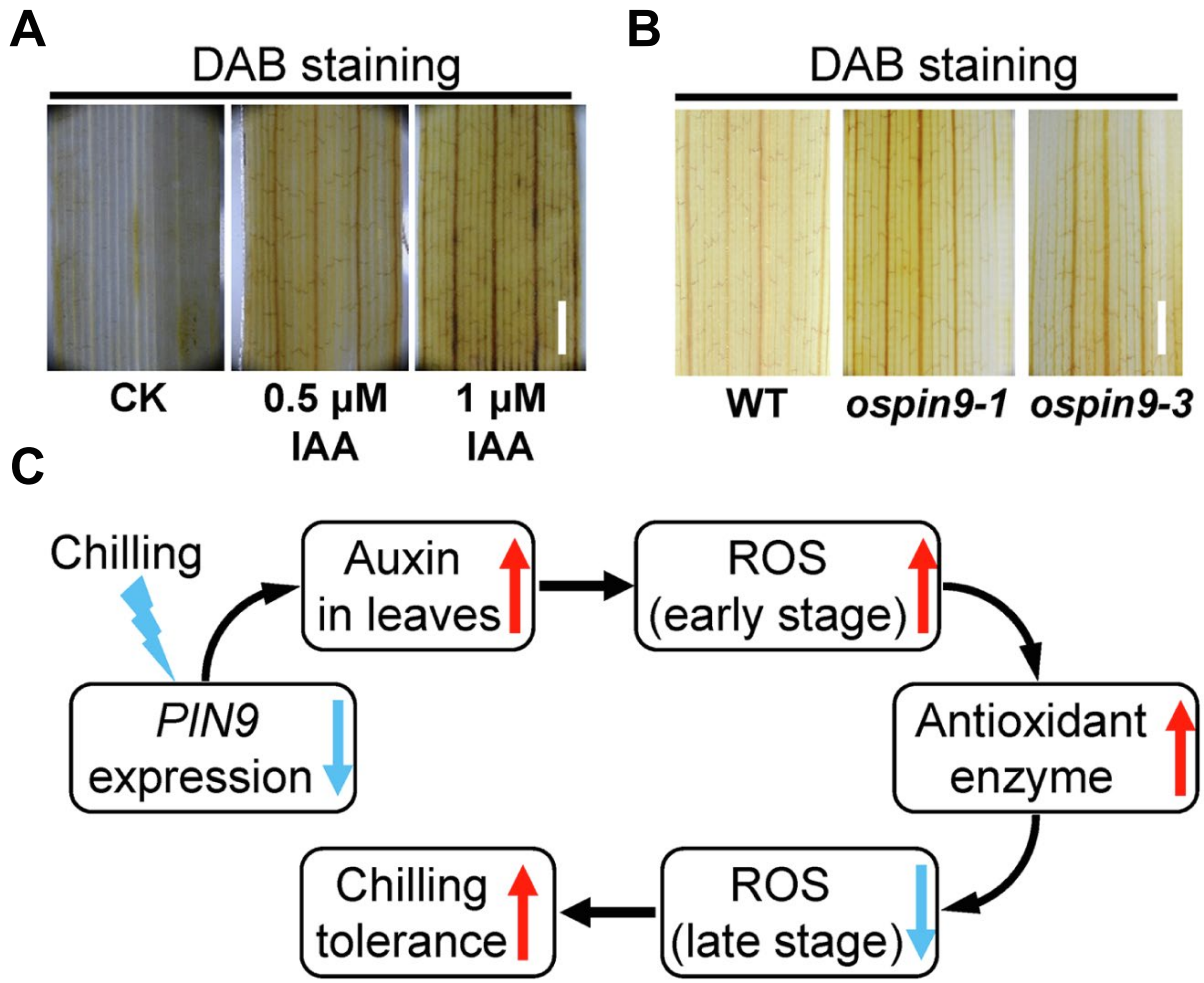
Effects of auxin application on the production of H_2_O_2_ and the proposed model of *OsPIN9*-mediated chilling tolerance in rice. **(A)** H_2_O_2_ content analysis after auxin application. Bar = 1 mm. **(B)** H_2_O_2_ content in WT and *ospin9* leaves under normal conditions. Bar = 1 mm. **(C)** Proposed model of *OsPIN9*-mediated chilling tolerance in rice.

It was reported that NAA treatment can induce the production of H_2_O_2_, indicating that auxin probably plays a crucial role in ROS homeostasis (Du et al., 2012). Therefore, we detected the H_2_O_2_ content in WT plants under indole-3-acetic acid (IAA) treatment by DAB staining. As shown in Figure 8A, the application of 0.5 μM and 1 μM IAA obviously accelerated the production of H_2_O_2_. Since *OsPIN9* functions in the transport of auxin from the main stem to the junction, and mutation of *OsPIN9* causes the accumulation of auxin in the leaves (Hou et al., 2021), so we further detected the H_2_O_2_ content in *ospin9* mutants under normal conditions. The results show that even under normal conditions, *ospin9* leaves showed a slight accumulation of H_2_O_2_ than WT plants (Figure 8B).

## Discussion

Low temperature is a major factor affecting rice growth and development. Over the past decades, many genes have been identified and demonstrated to be functional in cold adaption. The phytohormones, such as brassinosteroid (BR), abscisic acid (ABA), ethylene, and jasmonic acid (JA) also play a critical role in plant cold tolerance (Ding et al., 2020). Auxin, the first hormone discovered in plants, is one of the most important compounds in modulating plant growth and development, its role in regulating plant chilling tolerance is still largely unknown. As one of the monocot-specific *OsPIN* genes, *OsPIN9* is mainly expressed in adventitious root primordia and pericycle cells on stem bases (Wang et al., 2009; Miyashita et al., 2010), implying that OsPIN9 protein may function in regulating adventitious root development (Wang et al., 2009). Consistent with this speculation, Hou et al. (2021) reported that *OsPIN9* is positively involved in regulating adventitious root number in rice, as well as rice tiller number (Hou et al., 2021). Additionally, *OsPIN9* was reported to respond to heavy metal stresses, such as arsenic (Singh et al., 2017) and cadmium stress (Wang et al., 2021a). Despite recent progress in elucidating the biological function of *OsPIN9*, it is still at a beginning stage, especially for the function of *OsPIN9* in the regulation of abiotic stresses. In this report, we revealed that *OsPIN9* could be inhibited quickly by chilling stress and firstly demonstrated that *OsPIN9* is involved in chilling stress alleviation in rice by CRISPR/Cas9 technology at the seedling stage.

Rice is mainly distributed in tropical and subtropical areas and is sensitive to chilling stress (Kovach et al., 2007; Sang and Ge, 2007; Saito et al., 2010; Ma et al., 2015). Chilling stress severely impacts rice growth and development, and severe chilling disasters significantly impair rice yield (Zhang et al., 2019b). Improving chilling tolerance has great potential for improving rice yield under low-temperature conditions (Zhang et al., 2019b). Previous studies have reported that auxin transport is associated with chilling stress (Nadella et al., 2006; Shibasaki et al., 2009; Du et al., 2013a; Ashraf and Rahman, 2019), but there was no report regarding the role of OsPIN proteins in the regulation of chilling stress. In the present study, we functionally characterized the role of *OsPIN9* in chilling tolerance. Several lines of evidence support the result that *ospin9* mutants are more tolerant to chilling stress than WT plants. First, the survival rate of *ospin9* mutants was significantly higher than that of WT after chilling treatment (Figure 4). Second, as evidenced by trypan blue staining, the cell death in WT plants was more severe than that in *ospin9* mutants under chilling treatment (Figure 5A). Third, the electrolyte leakage, an index of cell membrane integrity, was significantly lower in *ospin9* mutants than in WT plants during chilling treatment (Figure 5B). Finally, MDA content, an indicator of lipid peroxidation, also showed a lower level in *ospin9* mutants than in WT plants after chilling treatment (Figure 5C). Collectively, these results strongly demonstrate that mutation of *OsPIN9* indeed increases the chilling tolerance in rice.

Plants have evolved multiple mechanisms to withstand chilling stress, among which cold-responsive transcription factors and their target genes, as well as ROS-dominated adaptation mechanism, have been demonstrated to play a critical role in the regulation of chilling stress (Yamaguchi-Shinozaki and Shinozaki, 2006; Zhang et al., 2016, 2019b). For example, it has been demonstrated that ICE1 and their target genes, such as *CBF*/*DREB* and *TPP1*, play a vital role in chilling resistance (Chinnusamy et al., 2003; Zhang et al., 2017). Although cold induces *OsDREB1* homologs (Dubouzet et al., 2003; Mao and Chen, 2012), overexpressing *OsDREB1A*, *OsDREB1B*, and *OsDREB1G* enhanced cold tolerance (Dubouzet et al., 2003; Ito et al., 2006; Moon et al., 2019), other reports showed that the expression levels of these transcription factors were not in line with the degree of cold tolerance (Mao and Chen, 2012; Zhao et al., 2015; Zhang et al., 2016). Our results showed that the expressions of *CBF*/*DREB* (*OsDREB1A*, *OsDREB1B*, *OsDREB1C*), *OsTPP1,* and Ca^2+^ signaling-related genes (*COLD1* and *OsCNGC9*) in *ospin9* mutants were all significantly higher than that in WT plants under normal conditions (Figure 6), and *ospin9* mutants accumulated more H_2_O_2_ than WT under normal conditions (Figure 8B). Since *OsDREB1* homologs could be induced by various abiotic stresses (Dubouzet et al., 2003; Moon et al., 2019), and Ca^2+^ influx into the cytoplasm can be triggered by ROS (Kwak et al., 2003), it is reasonable to speculate that defense responses in *ospin9* mutants have probably been triggered in advance even under normal conditions, and this probably partially alleviated the chilling stress in *ospin9* mutants. By contrast, the expression of *CBF*/*DREB* genes and *OsTPP1* in *ospin9* mutants was similar to or even lower than that in WT under chilling stress (Figures 6A,B), indicating that the CBF/DREB regulon probably plays a minor role in improving chilling tolerance of *ospin9* mutants, especially under chilling conditions. Although Ca^2+^ signaling genes *COLD1* and *OsCNGC9* were also decreased after chilling for 8 h in *ospin9* mutants (Figure 6C), Ca^2+^ influx into the cytosol is an early signaling event in response to stress stimuli in plants, which can be activated within seconds or minutes (Ma et al., 2015; Wang et al., 2021b). So whether Ca^2+^ signaling contributes to the enhanced chilling tolerance in *ospin9* mutants still needs further investigation.

Reactive oxygen species homeostasis is vital for plant growth and development. The plant needs to control ROS production whenever necessary precisely. Enzymatic and non-enzymatic antioxidant defense systems scavenge ROS and protect plant cells from oxidative damage (Mittler, 2002; Apel and Hirt, 2004). Chilling stress induces the production of H_2_O_2_ in the cell (Prasad et al., 1994b), and H_2_O_2_ level has dual effects during abiotic stress, i.e., low-level H_2_O_2_ at the early stress stage is considered as a signal to trigger stress-response, while high-level H_2_O_2_ at the later stress stage is considered as oxidative molecules to cause plant cell damage (Prasad et al., 1994b; Zhang et al., 2019b). The previous report showed that exogenous application of H_2_O_2_ can improve maize seedling chilling tolerance (Prasad et al., 1994a), and chilling acclimation indeed increased H_2_O_2_ content and chilling capacity in maize seedlings (Prasad et al., 1994b), indicating that proper H_2_O_2_ content can substantially enhance plant chilling tolerance. Consistent with this view, Zhang et al. (2016) demonstrated that *japonica* varieties with higher chilling tolerance accumulate more and less ROS at the middle and late chilling stages, respectively, while *indica* varieties which are more sensitive to chilling treatment performed inversely, indicating that ROS accumulation at different chilling stages plays different roles during chilling treatment. In line with these results, our study showed that *ospin9* mutants accumulated more and less ROS than WT at the early (36 h) and later (72 h) chilling stages, respectively, which was similar to the observation in *japonica* and *indica* varieties. Furthermore, the antioxidant enzyme system, including CAT, POD, and SOD, was all significantly enhanced in *ospin9* mutants than in WT plants at the early chilling stage (Figures 7D,E). Thus, we speculate that the ROS-dominated adaption mechanism plays a vital role in regulating *ospin9* chilling tolerance. Whether selection has acted on *OsPIN9*, just like *COLD1* (Ma et al., 2015), and contributed to the diversity of chilling tolerance between *japonica* and *indica* still needs further investigation.

Despite the crucial role of ROS homeostasis in modulating chilling tolerance in rice, the role of CBF/DREB regulon and Ca^2+^ signaling under chilling stress cannot be excluded in *ospin9* mutants. Early signaling events, including cytosolic Ca^2+^ influx and burst of ROS, can be activated within a very short time (Baxter et al., 2014). The interconnection of Ca^2+^ signaling with ROS production complicates the underlying mechanism under different abiotic stresses in plants, which are intimately correlated with diverse abiotic stresses (Baxter et al., 2014; Dong et al., 2022). Previous data showed that Ca^2+^ signal formation and H_2_O_2_ accumulation are fine-tuned in a reciprocal manner (Steinhorst and Kudla, 2013). Ca^2+^ signaling genes were significantly induced under normal conditions in *ospin9* mutants (Figure 6C), implying the probability of the enhanced cytosolic Ca^2+^ influx. Disruption of *OsPIN9* obviously enhanced the accumulation of H_2_O_2_ in *ospin9* mutant leaves (Figure 8B). The accumulated H_2_O_2_ and probably increased Ca^2+^ influx might trigger the defense response in advance in *ospin9* mutants, as evidenced by the increased expression of *OsDREB1s* and *OsTPP1* (Figures 6A,B), which may also contribute to the enhanced chilling tolerance in *ospin9* mutants. While the interconnection of Ca^2+^ and H_2_O_2_ signaling involved in chilling stress response still needs further investigation.

Previously, we have reported that *OsPIN9* is suppressed by chilling treatment, while induced by salt and drought treatments as well as ABA, gibberellin (GA), methyl jasmonate (MeJA), and salicylic acid (SA) treatments (Xu et al., 2022). Additionally, *cis*-element analysis of *OsPIN9* promoter also showed that the *OsPIN9* promoter contains many *cis*-elements that are predicted to be responsible to auxin, ABA, GA, MeJA, and SA (Xu et al., 2022), implying that *OsPIN9* might have a role under diverse abiotic stresses and be implicated in the interconnection of diverse phytohormones by influencing auxin homeostasis.

Auxin transport from shoot to root is mainly mediated by OsPIN9 and mutation of *OsPIN9* results in the accumulation of auxin in rice leaves (Hou et al., 2021). It is reasonable to speculate that more auxin is accumulated in *ospin9* mutants, which was further verified by NAA and NPA treatment assay (Figure 3), and more H_2_O_2_ accumulation was induced by auxin in leaves (Figure 8A). We speculated that rice probably possesses a native PAT-related chilling tolerance mechanism (Figure 8C). In brief, *OsPIN9* expression is quickly suppressed by low temperature (Figure 1), which results in the accumulation of auxin in leaves (Figure 3; Du et al., 2013a; Hou et al., 2021). The accumulated auxin promotes the production of H_2_O_2_ at the early chilling stress stage (Figure 7A), and H_2_O_2_ can be employed as a signal to trigger stress-responsive activities (Zhang et al., 2019b). After prolonged chilling treatment, improved antioxidant enzymes (Figures 7D,E) lead to reduced ROS (Figure 7B), which attenuates the damages caused by ROS and finally enhances chilling tolerance. Considering that mutation of *OsPIN9* substantially perturbed auxin homeostasis, caused growth retardation (Supplementary Figure 1; Hou et al., 2021), as well as enhanced chilling tolerance, chilling-induced quickly suppression of *OsPIN9* is likely to be an adaptive strategy that coordinately regulates growth rate and chilling tolerance in rice. In future, whether other cold-sensitive monocot plants, such as maize (*Zea mays*) and sorghum (*Sorghum bicolor*), also possess a similar chilling-resistant mechanism needs to be further investigated. Our results shed light on the relationship between PAT and chilling tolerance in rice.

Collectively, we investigated the role of *OsPIN9* in the regulation of chilling stress in rice in the present study. Although *ospin9* mutants show adverse effects on some agronomic traits, *OsPIN9* substantially contributes rice to survival under low-temperature stress. Further, investigation on how *OsPIN9* is rapidly inhibited after chilling treatment and ROS homeostasis is regulated by auxin homeostasis under normal and chilling conditions will improve our understanding of the mechanism of chilling stress in rice. Our discovery demonstrates that *OsPIN9* expression is negatively regulated by chilling stress and *OsPIN9* plays a critical role in chilling tolerance by modulating ROS homeostasis in rice.

## Data availability statement

The raw data supporting the conclusions of this article will be made available by the authors, without undue reservation.

## Author contributions

HX and CZ designed the experiments. XY, YZ, HW, SW, ZZ, and HX carried out the experiments. HL, HX, and XY analyzed the data and took photographs. HX wrote the article. HL, GA, and CZ revised and approved the article. All authors contributed to the article and approved the submitted version.

## Funding

This work was supported by the National Natural Science Foundation of China (3110019), the Natural Science Foundation of Henan province (182300410012), and the Student Research Training Program of Henan University of Science and Technology (2022452).

## Conflict of interest

The authors declare that the research was conducted in the absence of any commercial or financial relationships that could be construed as a potential conflict of interest.

## Publisher’s note

All claims expressed in this article are solely those of the authors and do not necessarily represent those of their affiliated organizations, or those of the publisher, the editors and the reviewers. Any product that may be evaluated in this article, or claim that may be made by its manufacturer, is not guaranteed or endorsed by the publisher.

## Footnotes

^1^ https://skl.scau.edu.cn/

## Supplementary material

Supplementary Figure 1 Comparison of phenotype between WT and *ospin9* mutants in rice at the seedling stage. **(A)** Phenotype of WT and *ospin9* mutants after germination for 7 days. Bar = 3 cm. **(B)** Phenotype of WT and *ospin9* mutants after germination for 14 days. Bar = 3 cm. Values are means ± standard deviation (SD; *n* = 12). Data were analyzed by ANOVA and Tukey’s tests at *p* < 0.05 significant level.
