# Supplementary figures and images for: CRISPR/Cas9-mediated mutation in auxin efflux carrier OsPIN9 confers chilling tolerance by modulating reactive oxygen species homeostasis in rice
Source: Front Plant Sci. 2022 Aug 1;13:967031. doi: 10.3389/fpls.2022.967031 (PMC9376474; doi:10.3389/fpls.2022.967031)

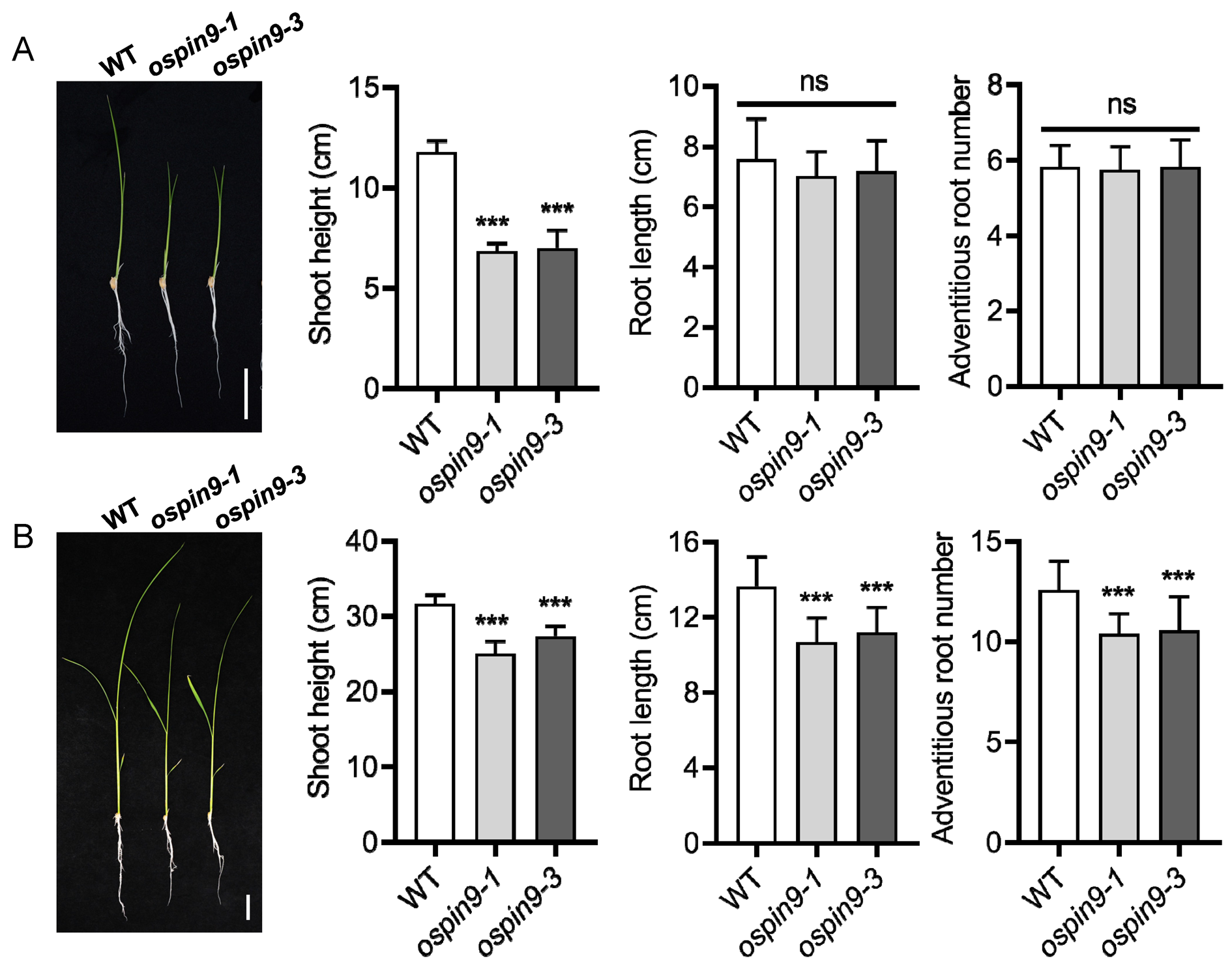

Supplement: Supplementary Figure 1 — Comparison of phenotype between WT and ospin9 mutants in rice at the seedling stage. (A) Phenotype of WT and ospin9 mutants after germination for 7 days. Bar = 3 cm. (B) Phenotype of WT and ospin9 mutants after germination for 14 days. Bar = 3 cm. Values are means ± standard deviation (SD; n = 12). Data were analyzed by ANOVA and Tukey’s tests at p < 0.05 significant level. [file Image_1.TIFF]
