# Supplementary material for: CRISPR/Cas9-mediated mutation in auxin efflux carrier OsPIN9 confers chilling tolerance by modulating reactive oxygen species homeostasis in rice
Source: Front Plant Sci. 2022 Aug 1;13:967031. doi: 10.3389/fpls.2022.967031 (PMC9376474; doi:10.3389/fpls.2022.967031)
Supplement: Supplementary file 2 [file Table_1.DOCX]

Table S1 Primers used in this study.

| **Primers for qRT-PCR** | Primer sequences (5’-3’) |
| --- | --- |
| OsPIN9-qF | GAGGACTCTCTGTTCACCATTC |
| OsPIN9-qR | GAGAACGACGCTATCTTGTATCC |
| OsYUC2-qF | TATGGATCGGCAACCATTTGA |
| OsYUC2-qR | CGCTGGGAAGACTGTCCTTGT |
| OsYUC3-qF | GGAAGCGTGTTCTCGTTGTTG |
| OsYUC3-qR | ACATTGACAGCCCAAAGGTGG |
| OsYUC4-qF | CCTCGACCTCTGCAACCACAATG |
| OsYUC4-qR | CGACAACAGGAGTACCAGCCAATC |
| OsYUC5-qF | GTCAGCCTCGACCTCTGCAACA |
| OsYUC5-qR | TGGGAAACCACTTGAGAAGGAACAC |
| OsYUC6-qF | GGATACCAAAGCAACGTCCCC |
| OsYUC6-qR | TGAAGCCAACAGAGTAGAGCCCTG |
| OsYUC7-qF | ACCGGCTACCGCAGCAATGTG |
| OsYUC7-qR | CGTACAGCCCCGACTCACCCT |
| OsDREB1A-qF | AGCGACCTGGCGTTCG |
| OsDREB1A-qR | TCGCGTAGTACAGGTCCCA |
| OsDREB1B-qF | GAGACCTTCGCCAACGATG |
| OsDREB1B-qR | CACCGGCAACACGTCCTT |
| OsDREB1C-qF | TACGGCAACATGGACTTCGA |
| OsDREB1C-qR | GCCCATCCCGTCGTAGTAGTAG |
| OsTPP1-qF | TGTCTCCCGTGATGAGAGCTG |
| OsTPP1-qR | AAACACCTTATTGCGGGACCTT |
| OsCNGC9-qF | GTGCTGTTTCTGCTCCATTTC |
| OsCNGC9-qR | TGCACTTGTCTGAAGAGGATTT |
| COLD1-qF | CAGGATATCAAAAGCTTGGATG |
| COLD1-qR | GCAGCTATCTTTGCTTGACG |
| OsACTIN1-qF | CTTCATAGGAATGGAAGCTGCG |
| OsACTIN1-qR | CACCTTGATCTTCATGCTGCTA |
| **Primers for mutant identification** |  |
| PIN9-Assay-F | CGACCTGGCTTACGAACGAA |
| PIN9-Assay-R | CCATGTCGAAGATGAGCACC |
| **Primers for off-target analysis** |  |
| 8500-F | GCTGATTTGTGGGGGTGTTGATGTG |
| 8500-R | ACTTCTTGCTTGCTTGGCGTGCTG |
